# The Frontal and Cerebellar Metabolism Related to Cognitive Dysfunction in Multiple System Atrophy

**DOI:** 10.3389/fnagi.2022.788166

**Published:** 2022-02-10

**Authors:** Cong Shen, Qi-Si Chen, Chuan-Tao Zuo, Feng-Tao Liu, Jian Wang

**Affiliations:** ^1^Department of Neurology and National Research Center for Aging and Medicine & National Center for Neurological Disorders, State Key Laboratory of Medical Neurobiology, Huashan Hospital, Fudan University, Shanghai, China; ^2^Positron Emission Tomography (PET) Center at Huashan Hospital, Institute of Functional and Molecular Medical Imaging, Human Phenome Institute, Fudan University, Shanghai, China

**Keywords:** cognitive impairment, ^18^F-fluorodeoxyglucose, fronto-striatal dopamine signaling, cerebellum, multiple system atrophy

## Abstract

**Background:**

Cognitive dysfunctions have been reported in multiple system atrophy (MSA). However the underlying mechanisms remain to be elucidated. This study aimed to explore the possible cerebral metabolism associated with domain-specific cognitive performances in MSA.

**Methods:**

A total of 84 patients were diagnosed as probable or possible MSA, comprised of 27 patients as MSA with predominant parkinsonism (MSA-P) and 57 patients as MSA with predominant cerebellar ataxia (MSA-C). The comprehensive neuropsychological tests and ^18^F-fluorodeoxyglucose (^18^F-FDG) positron emission tomography (PET) imaging were performed. *Z*-score was calculated to non-dimensionalize and unify indicators of different tests in the domains of executive function, attention, language, memory, and visuospatial function. Correlations between specific *Z*-score and cerebral ^18^F-FDG uptake were analyzed using statistical parametric mapping. The cognition-related metabolic differences between patients with MSA-P and MSA-C were analyzed using the *post-hoc* test.

**Results:**

*Z*-scores of the domains including attention, executive function, and language correlated positively with the metabolism in the superior/inferior frontal gyrus and cerebellum, but negatively with that in the insula and fusiform gyrus (*p* < 0.001). No significant differences in neuropsychological performances and frontal metabolism were found between patients with MSA-P and MSA-C. Only lower metabolism in the cerebellum was observed in MSA-C.

**Conclusion:**

Metabolic changes in the frontal lobe and cerebellum may participate in the cognitive impairments of patients with MSA. Nevertheless, cognitive and corresponding metabolic differences between the two subtypes of MSA still need more exploration.

## Introduction

Multiple system atrophy (MSA) is a sporadic, adult-onset neurodegenerative disorder, with the classical clinical presence of progressive autonomic failure, parkinsonism, and/or cerebellar ataxia (Wenning et al., 1997; Stefanova et al., 2009; Fanciulli and Wenning, 2015). Although dementia is a non-supporting item according to the second consensus statement on the diagnosis of MSA (Gilman et al., 2008), accumulating data showed great evidence for possible cognitive decline in MSA. Specifically, nearly half of the patients with MSA presented with cognitive impairments in some epidemiological data, with a broad spectrum of injury (Brown et al., 2010; Stankovic et al., 2014; Shen et al., 2021). At present, however, the underlying mechanisms of cognitive impairments in MSA are still far from being clearly understood. In the study of patients with MSA with mild dementia and aphasia, atrophy and neuronal cell loss were observed macroscopically and microscopically in the frontal lobe (Konagaya et al., 1999). Subsequent studies have compared the pathological burden degrees of glial cytoplasmic inclusions (GCIs) and neuronal cytoplasmic inclusions (NCIs) between MSA with normal cognition (MSA-NC) and MSA with cognitive impairment (MSA-CI; Cykowski et al., 2015; Homma et al., 2016; Koga et al., 2017; Jellinger, 2020; Miki et al., 2020). However, these results were variable and uncertain, which might be partially attributed to the small sample size.

The ^18^F-fluorodeoxyglucose (^18^F-FDG) positron emission tomography (PET) imaging can reflect the cerebral metabolic changes and disclose disease-specific alterations (Eckert et al., 2005, 2008). In our previous report, MSA-CI exhibited lower glucose metabolism in the left middle and superior frontal lobe, compared with MSA-NC (Shen et al., 2021). Nevertheless, the cerebral metabolic changes related to detailed cognitive performances in MSA remain to be further explored. In addition, the previous studies on comparisons of the profile and severity of cognitive dysfunction between two MSA subtypes, MSA with predominant parkinsonism (MSA-P) and MSA with predominant cerebellar ataxia (MSA-C; Gilman et al., 2008) remained limited and controversial (Kawai et al., 2008; Chang et al., 2009; Santangelo et al., 2020). Therefore, the comparison of cognition-related cerebral metabolism in different MSA subtypes will be helpful to understand the intrinsic pathological characteristics, which has not been reported earlier.

In this study, we aimed to detect the underlying cerebral metabolic changes by ^18^F-FDG PET imaging related to domain-specific cognitive dysfunctions in patients with MSA. Furthermore, we compared the neuropsychological performances and cognition-related brain metabolism between the two subtypes of MSA preliminarily.

## Participants and Methods

### Subjects

A total of 84 patients diagnosed with probable or possible MSA were recruited in the Department of Neurology, Huashan Hospital, Fudan University between February 2012 and May 2019. The clinical diagnosis and categorization between MSA-P and MSA-C were made according to the second consensus statement on the diagnosis of MSA published in 2008 (Gilman et al., 2008). Demographic information including age, age at onset, sex, disease duration, and education degree were systematically collected. All patients included neither had head injury nor had other neurological or psychiatric diseases.

This study was approved by the Human Studies Institutional Review Board, Huashan Hospital, Fudan University. Written informed consents were obtained from the participants before entering this study in accordance with the Declaration of Helsinki.

### Motor and Neuropsychological Assessments

Motor disability was evaluated by the Unified Parkinson’s Disease Rating Scale III (UPDRS III) in all participants in a Med-Off state, which means that the patients were off anti-parkinsonism drugs for more than 12 h. Global cognitive status and cognitive dysfunction in five domains covering attention, executive function, memory, visuospatial function, and language were assessed using Mini-Mental State Examination (MMSE) and a series of comprehensive neuropsychological tests, which were as follows: attention by the Symbol Digit Modalities Test (SDMT; Sheridan et al., 2006) and part A of Trail-Making Test (TMT-A; Zhao et al., 2013); executive function by the part C of Stroop Color-Word Test (CWT-C; Steinberg et al., 2005) and part B of Trail-Making Test (TMT-B; Zhao et al., 2013); memory function by the Auditory Verbal Learning Test (AVLT; Guo et al., 2009) and delayed recall task of the Rey-Osterrieth Complex Figure Test (CFT-delay recall) (Caffarra et al., 2002); visuospatial function by copy task of Rey-Osterrieth Complex Figure Test (CFT; Caffarra et al., 2002) and the Clock Drawing Test (CDT; Ricci et al., 2016); and language ability by Animal Verbal Fluency Test (AVFT) and Boston Naming Test (BNT; Lucas et al., 2005). To eliminate dimensional differences in tests and quantify impairments of cognition, neuropsychological results of each test of all patients were transformed into *Z*-score using the mean and SD of the norm data from healthy controls of similar age and education in the Shanghai area (Wu et al., 2018; Shen et al., 2021): *Z*-score = (test score - mean score of norm data)/SD of norm data (when data was a score) or *Z*-score = (mean time of norm data - test time)/SD of norm data (when data were a time metrics) (Wu et al., 2018). *Z*-score less than −1.5 was defined to be the impairment in each test and *Z*-score of each cognition domain was calculated using the average *Z*-score of two tests in the same cognition domain.

### ^18^F-Fluorodeoxyglucose Positron Emission Tomography Scan and Image Processing

^18^F-fluorodeoxyglucose PET imaging was acquired in all patients within 1 month before or after the clinical assessment in the Huashan Hospital. Patients were instructed to be fasting for at least 6 h in an off-drug state before the scan. Forty-five minutes resting after injection of ^18^F-FDG (185 MBq), a short CT scan, and subsequent 10-min PET scan was performed by a Siemens Biograph 64 PET/CT (Siemens, Munich, Germany) in the three-dimensional (3D) mode in a dimly lit and quiet room. Images were subsequently reconstructed using a 3D ordered-subset expectation-maximization algorithm (4 iterations; 24 subsets; Gaussian filter, 2 mm; zoom, 3). Reconstructed images had a matrix size of 168 × 168 × 148 and a voxel size of 2 × 2 × 1.5 mm^3^.

Positron emission tomography images were transformed for further processing using the MRIcron tool (output format: hdr/img). Then, images were processed by statistical parametric mapping (SPM5) software operated in MATLAB platform (Mathworks Inc., Sherborn, MA, United States). First, the original images were spatially normalized to normative stereotactic Montreal Neurological Institute (MNI) space using default [^15^O]-H2O PET template (Della Rosa et al., 2014)^1^. Second, they were smoothed through a three-dimensional Gaussian filter with 10-mm full-width at half-maximum (FWHM).

To compare the metabolic characteristics between MSA-P and MSA-C, the two-sample *t*-test was used with age, sex, disease duration, and UPDRS score as covariates according to the general linear model at each voxel in SPM. To assess whether metabolic characteristics in a specific brain region would correlate with *Z*-score in each cognitive domain, the multiple regression model established by us previously (Ge et al., 2015; Wu et al., 2018) was applied first to detect clusters with peak threshold set at *P* < 0.001 over whole brain regions and extent threshold set as 120 voxels (960 mm^3^) and further to highlight clusters survived after a false discovery rate (FDR) correction at *p* < 0.05. In this step, education degree and UPDRS III score were both included as covariates to eliminate the effect of these factors. The differences in global metabolic values were modeled by analysis of covariance (ANCOVA) to minimize intersubject variability and improve signal-noise ratios. Exact coordinates and corresponding brain regions of detected clusters were ascertained through an MNI-to-Talairach conversion using Talairach–Daemon software (Research Imaging Center, University of Texas Health Science Center, San Antonio, TX, United States). The SPM maps for positive and negative correlations were superpositioned on a standard T1-weighted MRI brain template. Then, the spherical volume of interest (VOI) (4 mm radius) with the center of the circle at the peak voxel of the cluster was constructed, and metabolic value in each VOI was quantified using ScAnVP software (Version 5.9.1; Center for Neuroscience, the Feinstein Institute for Medical Research, Manhasset, NY, United States). To eliminate the individual differences in global metabolic value, the normalized regional cerebral metabolic rate of glucose (rCMRglc) was calculated by the following formula: [VOI value/whole-brain metabolism] × 100%.

### Statistical Analysis

Differences in the demographic information, clinical characteristics, and neuropsychological performances between MSA-P and MSA-C were analyzed through Student’s *t*-test in continuous and normally distributed data. Pearson’s χ^2^ test was used in categorical data. Mann–Whitney U test was used in continuous and non-parametric data. The *post-hoc* correlation analysis between *Z*-score in the specific cognitive domain and normalized rCMRglc in each VOI was performed through Pearson correlation and linear regression analyses. In the *post-hoc* metabolic comparison between patients with MSA-P and MSA-C, multiple comparisons *via* the Benjamini–Hochberg procedure were performed to control the FDR. Statistical analyses were performed using SPSS software (SPSS for Windows, version 22.0; SPSS Inc., Chicago, IL, United States). *p* < 0.05 was considered statistically significant.

## Results

### Clinical Characteristics and Neuropsychological Performances in Patients With Multiple System Atrophy

Demographic, clinical, and detailed neuropsychological information of the total 84 patients with MSA was depicted in Table 1. The mean age at onset was 54.89 ± 7.97 years, and the mean disease duration was 26.80 ± 20.69 months. Patients with MSA were divided into two subtypes according to their predominant clinical symptoms, including 27 patients with MSA-P and 57 patients with MSA-C. No group differences were found for age at onset and education, but differences were observed in age, sex, disease duration, and UPDRS III score (Table 1). Both subtypes had considerable and extensive cognitive impairments in each domain represented by number and percentage of impairment as follows: attention, MSA-P, 18(67%), MSA-C, 30(53%); executive function, MSA-P, 13(48%), MSA-C, 32(56%); memory, MSA-P, 18(67%), MSA-C, 25(44%); visuospatial function, MSA-P, 12(44%), MSA-C, 22(39%); language, MSA-P, 10(37%), MSA-C, 21(37%). Compared with patients with MSA-C, patients with MSA-P performed worse on the attention domain, judging by a lower *Z*-score of attention domain and two corresponding tests (SDMT and TMT-A) (*p* < 0.05). However, this difference in attention performance was not significant after adjusting age, sex, disease duration, and UPDRS score factors.

**TABLE 1:** Demographic, clinical characteristics, and neuropsychological performance in 84 patients with multiple system atrophy (MSA).

	Total MSA (*n* = 84)	MSA-P (*n* = 27)	MSA-C (*n* = 57)	*p*
Age (years)	57.12 (8.01)	59.89 (7.21)	55.81 (8.10)	0.028
Age at onset (years)	54.89 (7.97)	57.08 (7.14)	53.85 (8.19)	0.082
Sex (male)	51	11	40	0.010
MSA duration (months)	26.80 (20.69)	33.70 (25.50)	23.53 (17.28)	0.034
Education (years)	10.82 (3.19)	10.67 (3.33)	10.90 (3.14)	0.761
UPDRS III score_OFF	33.58 (15.42)	48.37 (15.80)	26.58 (9.02)	<0.001
**MMSE**	26.43 (2.80)	26.48 (2.41)	26.40 (2.98)	0.906
**Attention**	48(57%)	18(67%)	30(53%)	0.225
*Z* score	−1.58(1.62)	−2.28(1.90)	−1.24(1.37)	0.005
SDMT score	23.56 (13.09)	19.33 (12.36)	25.56 (13.04)	0.041
TMT-A time	90.73 (46.98)	111.19 (57.78)	81.04 (37.73)	0.018
**Executive function**	45(54%)	13(48%)	32(56%)	0.493
*Z* score	−1.10(1.18)	−1.10(1.32)	−1.09(1.12)	0.990
CWT-C score	41.30 (6.82)	43.26 (6.21)	40.37 (6.94)	0.069
TMT-B time	203.58 (80.81)	223.26 (101.01)	194.26 (68.31)	0.125
**Memory**	43(51%)	18(67%)	25(44%)	0.051
*Z* score	−1.12(1.10)	−1.32(1.10)	−1.03(1.09)	0.260
AVLT				
-Short delayed recall	3.99 (2.25)	3.63 (2.42)	4.16 (2.16)	0.317
-Long delayed recall	3.77 (2.22)	3.56 (2.52)	3.88 (2.09)	0.539
-cued recall	3.77 (2.42)	3.26 (2.47)	4.02 (2.38)	0.182
CFT-delay recall	11.71 (6.97)	10.30 (5.74)	12.39 (7.44)	0.201
**Visuospatial function**	34(40%)	12(44%)	22(39%)	0.610
*Z* score	−1.42(1.48)	−1.39(1.25)	−1.43(1.58)	0.897
CFT-copy score	27.74 (7.43)	26.93 (7.73)	28.12 (7.32)	0.494
CDT score	19.25 (7.10)	20.19 (6.82)	18.81 (7.24)	0.409
**Language**	31(37%)	10(37%)	21(37%)	0.986
*Z* score	−1.05(1.08)	−0.95(0.97)	−1.11(1.13)	0.534
AVFT score	13.37 (4.44)	13.15 (4.54)	13.47 (4.43)	0.756
BNT score	21.57 (4.37)	22.30 (4.26)	21.23 (4.42)	0.298

*Data are shown in mean (SD) or number (%) of impairment or N. p-value represents the significance level of t-test performed for each item between patients with MSA-P and MSA-C. MSA-P, MSA with predominant parkinsonism; MSA-C, MSA with predominant cerebellar ataxia; UPDRS, Unified Parkinson’s Disease Rating Scale; MMSE, Mini-Mental State Examination; SDMT, Symbol Digit Modalities Test; TMT, Trail Making Test; CWT-C, Stroop Color-Word Test C; AVLT, Auditory Verbal Learning Test; CFT, the Rey-Osterrieth Complex Figure Test; CDT, Clock Drawing Test; AVFT, Animal Verbal Fluency Test; BNT, Boston Naming Test.*

### The Regional Metabolism Correlating With Cognitive Declines in Multiple System Atrophy

**FIGURE 1:**
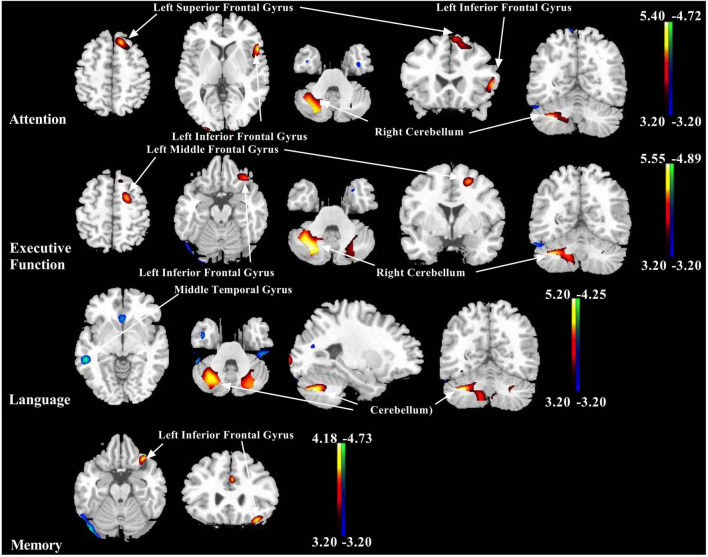
Representative images showing correlations between *Z*-score of each cognitive domain and ^18^F-fluorodeoxyglucose (^18^F-FDG) metabolism in patients with multiple system atrophy (MSA). Positive relationships are displayed with a red-yellow scale while negative relationships are displayed using a blue-green scale. Both are overlaid on a structural magnetic resonance image (MRI) brain template. White arrows show the representative brain regions. The thresholds of the color bars indicate *T*-values, and voxel threshold was set at *p* < 0.001.

Global metabolic values did not correlate with any *Z*-score in five domains (regression analysis: absolute value *r* ≤ 0.07, *p* ≥ 0.40). However, the positive and negative correlations between the regional ^18^F-FDG metabolism and the cognitive *Z*-score in the five domains were detected (Figure 1).

The brain regions correlated positively to the *Z-*score of the separate cognitive domain were described as follows, respectively: attention with left inferior frontal gyrus, left superior frontal gyrus, left medial frontal gyrus, and right cerebellum (uvula and cerebellar tonsil); executive function with left inferior frontal gyrus, left middle frontal gyrus, left cerebellum (uvula), and right cerebellum (pyramis and inferior semilunar lobule); language with left superior frontal gyrus, left middle frontal gyrus, left cerebellum (pyramis and uvula), and right cerebellum (tuber, pyramis, cerebellar tonsil, and declive); memory with inferior frontal gyrus; and visuospatial function with left cerebellum (pyramis) (Figure 1 and Table 2).

**TABLE 2:** Brain regions in the 84 patients with MSA exhibiting positive correlations between the *Z*-score of cognitive domains and regional brain metabolism adjusting for education and Unified Parkinson’s Disease Rating Scale (UPDRS) score.

Domains	Regions	BA	MNI coordinate			*Z* max	Cluster size (mm^3^)	Pearson correlation coefficient
			x	y	z			
Attention	Left Inferior Frontal Gyrus #	45	−56	18	4	4.97	2,936	0.5612
	Left Superior Frontal Gyrus	8	−14	28	52	4.43	3,184	0.4160
	Left Superior Frontal Gyrus	6	−8	24	58	3.94	3,184	0.4183
	Left Medial Frontal Gyrus	6	−6	42	40	3.23	3,184	0.2747
	Right Uvula (Cerebellum) #	/	30	−72	−36	4.55	3,600	0.3190
	Right Cerebellar Tonsil (Cerebellum)	/	14	−58	−42	3.71	3,600	0.1943
Executive function	Left Middle Frontal Gyrus #	6	−22	0	54	4.37	1,632	0.4857
	Left Inferior Frontal Gyrus	47	−36	30	−20	4.10	1,312	0.4578
	Left Uvula (Cerebellum)	/	−28	−68	−36	3.67	1,280	0.3073
	Right Pyramis (Cerebellum) #	/	28	−68	−38	5.09	5,296	0.4758
	Right Inferior Semi-Lunar Lobule (Cerebellum)	/	24	−64	−50	3.48	5,296	0.3136
Language	Left Middle Frontal Gyrus	11	−32	38	−20	3.60	1,064	0.2883
	Left Superior Frontal Gyrus	11	−38	46	−16	3.45	1,064	0.2330
	Left Pyramis (Cerebellum) #	/	−22	−74	−42	4.19	3,168	0.3997
	Left Uvula (Cerebellum) #	/	−30	−80	−36	4.46	3,168	0.3793
	Right Tuber (Cerebellum) #	/	34	−66	−36	4.81	5,208	0.4574
	Right Pyramis (Cerebellum) #	/	26	−72	−38	4.70	5,208	0.4547
	Right Cerebellar Tonsil	/	6	−56	−50	4.19	5,208	0.3801
	Right Declive (Cerebellum)	/	34	−66	−14	4.05	960	0.4508
Memory	Left Inferior Frontal Gyrus	47	−36	28	−22	3.75	1,000	0.4088
Visuospatial function	Left Pyramis (Cerebellum)	/	−28	−66	−36	3.60	1,160	0.3240

*Statistical threshold: p < 0.001. # indicates the cluster survived after FDR correction, p < 0.05. Pearson correlation coefficient evaluates correlations between Z-score in cognitive domain and normalized rCMRglc. BA, Brodmann area; MNI, Montreal Neurological Institute; and UPDRS, Unified Parkinson’s Disease Rating Scale III.*

However, the brain regions associated negatively with the *Z*-score of the separate cognitive domain were shown as follows, respectively: attention with right fusiform gyrus and right insula; executive function with right fusiform gyrus and right inferior temporal gyrus; language with left anterior cingulate, right middle temporal gyrus, and right fusiform gyrus; and memory with right fusiform gyrus and right inferior occipital gyrus (Figure 1 and Table 3).

**TABLE 3:** Brain regions in the 84 patients with MSA exhibiting negative correlations between the *Z*-score of cognitive domains and regional brain metabolism adjusting for education and UPDRS score.

Domain	Regions	BA	MNI coordinate			*Z* max	Cluster size (mm^3^)	Pearson correlation coefficient
			x	y	z			
Attention	Right Fusiform Gyrus	20	58	−34	−32	4.42	3,256	−0.4717
	Right Fusiform Gyrus	37	60	−52	−28	3.60	3,256	−0.3563
	Right Fusiform Gyrus	19	50	−72	−22	3.26	3,256	−0.2992
	Right Postcentral Gyrus	2	42	−28	32	3.92	3,032	−0.4284
	Right Insula	13	40	−10	26	3.80	3,032	−0.4841
	Right Insula	13	38	−22	28	3.60	3,032	−0.4676
Executive function	Right Fusiform Gyrus	20	56	−36	−32	4.56	4,464	−0.4900
	Right Inferior Temporal Gyrus	37	62	−48	−28	3.84	4,464	−0.4225
Language	Left Anterior Cingulate	24	0	24	−8	3.72	1,024	−0.3978
	Right Middle Temporal Gyrus	/	54	−38	−6	4.03	1,728	−0.4089
	Right Fusiform Gyrus	20	58	−42	−32	3.90	1,128	−0.3894
	Right Fusiform Gyrus	37	60	−52	−28	3.50	1,128	−0.3344
	Right Fusiform Gyrus	20	50	−30	−34	3.20	1,128	−0.3349
Memory	Right Fusiform Gyrus	19	46	−80	−22	4.43	2,824	−0.4503
	Right Inferior Occipital Gyrus	17	24	−100	−18	3.33	2,824	−0.3420

*Statistical threshold: p < 0.001. None of the clusters survived after FDR correction, p < 0.05. Pearson correlation coefficient evaluates correlations between Z-score in the cognitive domain and normalized rCMRglc. BA, Brodmann area; MNI, Montreal Neurological Institute.*

**FIGURE 2:**
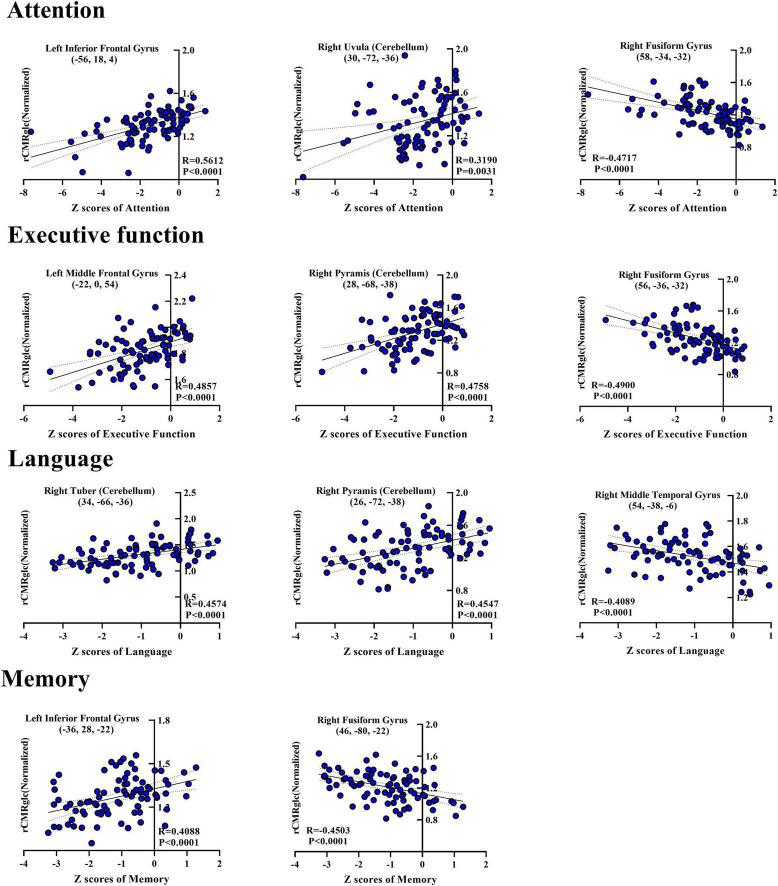
Scatterplots showing correlations between *Z*-score of each cognitive domain and normalized regional glucose metabolic activities for the significantly correlated regions in the *post-hoc* analysis. Each blue-filled circle represents one patient with *Z*-score of specific cognitive domain and regional cerebral metabolic rate of glucose (rCMRglc) in certain brain region. *R*-value means the correlation coefficients. rCMRglc, regional cerebral metabolic rate of glucose.

Moreover, the *post-hoc* correlations between the *Z*-score in separate cognition domain and the normalized rCMRglc in each significant brain region were calculated, with the correlation coefficients listed in Tables 2, 3. The scatterplots and relationships of cognition and metabolic value in 11 representative regions are shown in Figure 2.

### Cognition-Related Regional Metabolism in Two Subtypes of Multiple System Atrophy

Compared with patients with MSA-C, increased metabolism mainly in the bilateral cerebellum while decreased metabolism in bilateral putamen was observed in MSA-P, with no difference in frontal gyrus (Supplementary Table 1).

**FIGURE 3:**
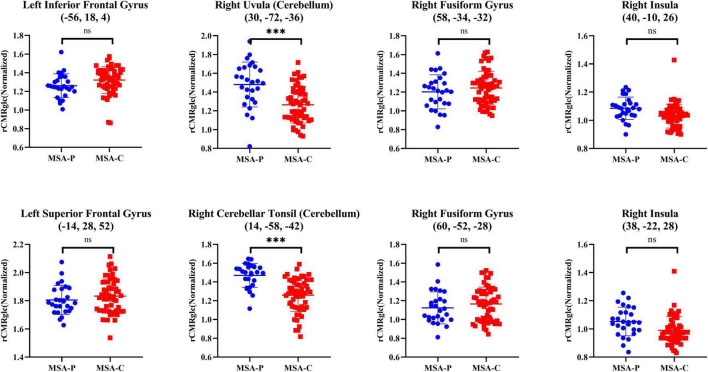
Differences of normalized rCMRglc in representative attention-related regions between two subtypes of patients with MSA. The error bars represent SD. ^***^p < 0.0001; *p < 0.01; *^ns^p* > 0.05. rCMRglc, regional cerebral metabolic rate of glucose; MSA-P, multiple system atrophy with predominant parkinsonism; MSA-C, multiple system atrophy with cerebellar ataxia.

Considering the unadjusted cognitive differences in the attention domain between MSA-P and MSA-C, the comparisons of regional metabolism in attention-related areas mentioned above between two subtypes are presented in Figure 3 and Table 4. However, no significant metabolic intergroup differences were observed in the left inferior frontal gyrus, superior frontal gyrus, medial frontal gyrus, right fusiform gyrus, right postcentral gyrus, and right insula. Only the metabolism in the right cerebellum (including uvula and cerebellar tonsil) was significantly lower in patients with MSA-C than patients with MSA-P after adjustment for confounders and *post-hoc* correction for multiple comparisons.

**TABLE 4:** Differences of regional brain metabolism in attention-related areas between patients with MSA with predominant parkinsonism (MSA-P) and MSA with predominant cerebellar ataxia (MSA-C).

Domains	Regions	BA	MNI coordinate			MSA-P	MSA-C	*p*	*p* ^a^
			*x*	*y*	*z*	(*n* = 27)	(*n* = 57)		
Attention	Left Inferior Frontal Gyrus	45	−56	18	4	1.26 (0.13)	1.32 (0.14)	0.065	0.959
	Left Superior Frontal Gyrus	8	−14	28	52	1.81 (0.10)	1.83 (0.12)	0.314	0.965
	Left Superior Frontal Gyrus	6	−8	24	58	1.79 (0.09)	1.82 (0.10)	0.175	0.981
	Left Medial Frontal Gyrus	6	−6	42	40	1.75 (0.08)	1.77 (0.09)	0.458	0.965
	Right Uvula (Cerebellum)	/	30	−72	−36	1.48 (0.24)	1.26 (0.19)	<0.0001	<0.0001
	Right Cerebellar Tonsil (Cerebellum)	/	14	−58	−42	1.47 (0.13)	1.26 (0.18)	<0.0001	<0.0001
	Right Fusiform Gyrus	20	58	−34	−32	1.20 (0.18)	1.24 (0.18)	0.325	0.055
	Right Fusiform Gyrus	37	60	−52	−28	1.12 (0.17)	1.17 (0.18)	0.315	0.055
	Right Fusiform Gyrus	19	50	−72	−22	1.29 (0.19)	1.31 (0.19)	0.532	0.115
	Right Postcentral Gyrus	2	42	−28	32	1.10 (0.10)	1.08 (0.09)	0.303	0.965
	Right Insula	13	40	−10	26	1.08 (0.08)	1.03 (0.08)	0.005	0.202
	Right Insula	13	38	−22	28	1.05 (0.10)	0.99 (0.10)	0.008	0.223

*Data are shown in mean (SD) of normalized rCMRglc in each attention-related region in patients with MSA-P and MSA-C. p-value represents the significance level of t-test performed for normalized rCMRglc in each attention-related region between patients with MSA-P and MSA-C. ^a^p-value represents the significance level for normalized rCMRglc in each attention-related region between patients with MSA-P and MSA-C after adjustment for age, sex, disease duration, and UPDRS score and application of the post-hoc correction for multiple comparisons. MSA-P, multiple system atrophy with predominant parkinsonism; MSA-C, multiple system atrophy with predominant cerebellar ataxia; BA, Brodmann area; MNI, Montreal Neurological Institute.*

In addition, correlations between metabolism and the cognitive *Z*-score in the five domains separately in two subtypes are shown in Supplementary Tables 2, 3. Cognition in patients with MSA-C correlated positively with metabolism in the frontal lobe and cerebellum, negatively with that in the fusiform gyrus and insula, whereas the clusters correlated with cognition in patients with MSA-P were limited to the cerebellum (positively) and temporal lobe (negatively).

## Discussion

In this study, there were three main findings. First and most importantly, lesions in the domains of attention, executive function, and language (*Z*-scores) correlated positively with the metabolism in the frontal gyrus and cerebellum and negatively with the insula and fusiform gyrus. Second, the attention function tended to be more severely impaired in MSA-P than in MSA-C, though significance did not sustain after adjusting confounders. Third, among all the attention-related brain regions, there was only lower metabolism of the cerebellum in MSA-C than in MSA-P.

^18^F-fluorodeoxyglucose PET imaging can detect cerebral glucose metabolism and offer us some hints for mechanisms in neural hubs or networks behind cognitive impairments in patients with MSA. In our analysis, metabolism in the frontal gyrus and cerebellum showed a great correlation with the severity of cognitive impairments in attention, executive function, and language domains.

The lower metabolism in the frontal gyrus of patients with MSA with worse cognition might suggest the disruption of frontostriatal dopamine signaling. The prefrontal cortex, as a key hub in the executive network, is associated with working memory and selective attention (Elliott, 2003; Gratwicke et al., 2015). Dopamine released from the substantia nigra and ventral tegmental area modulates prefrontal D2 receptors and facilitates cognitive flexibility (Floresco and Magyar, 2006). In previous neuropathology studies, there was a significant loss of neurons in the frontal cortex in patients with MSA with executive dysfunction compared with those with normal executive function (Salvesen et al., 2017). Analogously, (Fiorenzato et al., 2017) found that MSA-CI had focal volume reduction in the left middle frontal lobe compared with MSA-NC through voxel-based morphometry. Particularly, the study by Chang et al. (2009) showed that memory scores were well associated with prefrontal lobe atrophy. In addition, our hypothesis of striatofrontal differentiation in patients with MSA-CI was supported by the finding from FDG study by Kim et al. (2017), in which the glucose metabolism in the striatum was the most powerful determinant to metabolism in the frontal gyrus in the patients with MSA but not in healthy controls. Considering all these studies together, we believed that the frontostriatal network was involved in cognitive dysfunction in patients with MSA.

Besides the well-known motor function, the cerebellum is also involved in maintaining the functions of working memory, language, and social cognition. As previously reported, cerebellar lesions could lead to executive dysfunctions, deficits in visuospatial processing, and linguistic skills, termed cerebellar cognitive affective syndrome (Schmahmann and Sherman, 1998; Buckner, 2013). In study by Kawabata et al. (2019) with resting-state fMRI, it was reported that Addenbrooke’s Cognitive Examination-Revised scores correlated strongly with functional connectivity between the cerebellum and the medial prefrontal/anterior cingulate cortices. All these studies indicated extensive disruptions in fronto-cerebellar networks in patients with MSA. In this study, we found the reduced metabolism in the frontal lobe and cerebellum associated with severer cognitive injury, possibly supported by the role of cerebellum degeneration in cognitive impairments.

Furthermore, the cognitive impairments in MSA-P and MSA-C were evaluated and compared in this study. As previously reported, some studies suggested the patients with MSA-P showed more deteriorated cognitive functions (Kawai et al., 2008; Li et al., 2021), some found patients with MSA-C performing worse on specific cognitive tests (Chang et al., 2009; Barcelos et al., 2018), while the rest showed no difference (Siri et al., 2013; Santangelo et al., 2020). In our study, patients with MSA-P performed poorly in the domain of attention, to a weak degree that did not survive after adjusting confounding factors. Therefore, we could not make any definite conclusion on the differences in cognitive dysfunctions between two MSA subtypes so far.

Among attention-related brain regions in this study, we found regional metabolism in the superior/inferior/medial frontal gyrus showing no difference between MSA-P and MSA-C, but metabolism in the cerebellum was lower in MSA-C. Kawai et al. (2008) found cerebellar hypoperfusion in MSA-C, and hypoperfusion in right putamen in MSA-P when comparing brain perfusion between MSA-P and MSA-C. Given significant differences in motion spectrum between MSA-P and MSA-C, the metabolic pattern in putamen and cerebellum was essentially distinct. Therefore, there is not enough evidence to support whether hypermetabolism in the cerebellum in MSA-P could explain the unadjusted difference of attention performance between two types of MSA. More studies with rigorous design are needed to answer this question.

Our study had several strengths. First, our study sample size was relatively large compared with the previous reports. Second, the neuropsychological tests applied in this study were comprehensive to cover the five domains. However, several limitations should also be admitted. Partial volume effect (PVE) correction was not performed due to the incomplete data of MRI. In future studies, rigorous results performed based on PVE correction in newly recruited patients are warranted. Moreover, the demographic information of patients with MSA-P and MSA-C was mismatched, and the sample size of MSA-P was relatively small in our study. Further study with a more paired and more complete population is needed to confirm the neuropsychological and metabolic comparisons between the two groups. In addition, double-dipping problem causing selective bias was another limitation. It derived from performing MSA-P and MSA-C comparison after extracting metabolic values from statistically significant regions. We hope that another independent data set in the future could confirm our results.

## Conclusion

The frontal lobe and cerebellum participated in the pathogenesis of cognitive impairments in MSA in this ^18^F-FDG PET imaging study. Longitudinal studies with specific radioligands are needed to clarify the unique role of dopaminergic deficiency and misfolded protein burden in the development of cognition decline in MSA.

## Data Availability Statement

The raw data supporting the conclusions of this article will be made available by the authors, without undue reservation.

## Ethics Statement

The studies involving human participants were reviewed and approved by the Human Studies Institutional Review Board, Huashan Hospital, Fudan University. The patients/participants provided their written informed consent to participate in this study.

## Author Contributions

JW and F-TL were involved in the conception and design of the study. CS and Q-SC took part in the acquisition, screening, and analysis of data. CS was responsible for figures and tables. CS, Q-SC, and F-TL wrote the first draft of the manuscript. C-TZ, F-TL, and JW revised the manuscript. All authors contributed to the article and approved the submitted version.

## Conflict of Interest

The authors declare that the research was conducted in the absence of any commercial or financial relationships that could be construed as a potential conflict of interest.

## Publisher’s Note

All claims expressed in this article are solely those of the authors and do not necessarily represent those of their affiliated organizations, or those of the publisher, the editors and the reviewers. Any product that may be evaluated in this article, or claim that may be made by its manufacturer, is not guaranteed or endorsed by the publisher.

## Footnotes

1
www.fil.ion.ucl.ac.uk/spm/spm99.html

## References

[B1] Barcelos L. B. Saad F. Giacominelli C. Saba R. A. de Carvalho Aguiar P. M. Silva S. M. A. (2018). Neuropsychological and clinical heterogeneity of cognitive impairment in patients with multiple system atrophy. *Clin. Neurol. Neurosurg.* 164 121–126. 10.1016/j.clineuro.2017.10.039 29223069

[B2] Brown R. G. Lacomblez L. Landwehrmeyer B. G. Bak T. Uttner I. Dubois B. (2010). Cognitive impairment in patients with multiple system atrophy and progressive supranuclear palsy. *Brain* 133(Pt 8) 2382–2393. 10.1093/brain/awq158 20576697

[B3] Buckner R. L. (2013). The cerebellum and cognitive function: 25 years of insight from anatomy and neuroimaging. *Neuron* 80 807–815. 10.1016/j.neuron.2013.10.044 24183029

[B4] Caffarra P. Vezzadini G. Dieci F. Zonato F. Venneri A. (2002). Rey-Osterrieth complex figure: normative values in an Italian population sample. *Neurol. Sci.* 22 443–447. 10.1007/s100720200003 11976975

[B5] Chang C. C. Chang Y. Y. Chang W. N. Lee Y. C. Wang Y. L. Lui C. C. (2009). Cognitive deficits in multiple system atrophy correlate with frontal atrophy and disease duration. *Eur. J. Neurol.* 16 1144–1150. 10.1111/j.1468-1331.2009.02661.x 19486137

[B6] Cykowski M. D. Coon E. A. Powell S. Z. Jenkins S. M. Benarroch E. E. Low P. A. (2015). Expanding the spectrum of neuronal pathology in multiple system atrophy. *Brain* 138(Pt 8) 2293–2309. 10.1093/brain/awv114 25981961 PMC4840945

[B7] Della Rosa P. A. Cerami C. Gallivanone F. Prestia A. Caroli A. Castiglioni I. (2014). A standardized [18F]-xFDG-PET template for spatial normalization in statistical parametric mapping of dementia. *Neuroinformatics* 12 575–593. 10.1007/s12021-014-9235-4 24952892

[B8] Eckert T. Barnes A. Dhawan V. Frucht S. Gordon M. F. Feigin A. S. (2005). FDG PET in the differential diagnosis of parkinsonian disorders. *Neuroimage* 26 912–921. 10.1016/j.neuroimage.2005.03.012 15955501

[B9] Eckert T. Tang C. Ma Y. Brown N. Lin T. Frucht S. (2008). Abnormal metabolic networks in atypical parkinsonism. *Mov. Disord.* 23 727–733. 10.1002/mds.21933 18186116

[B10] Elliott R. (2003). Executive functions and their disorders. *Br. Med. Bull.* 65 49–59. 10.1093/bmb/65.1.49 12697616

[B11] Fanciulli A. Wenning G. K. (2015). Multiple-system atrophy. *N. Engl. J. Med.* 372 249–263. 10.1056/NEJMra1311488 25587949

[B12] Fiorenzato E. Weis L. Seppi K. Onofrj M. Cortelli P. Zanigni S. (2017). Brain structural profile of multiple system atrophy patients with cognitive impairment. *J. Neural Transm.* 124 293–302. 10.1007/s00702-016-1636-0 27778099

[B13] Floresco S. B. Magyar O. (2006). Mesocortical dopamine modulation of executive functions: beyond working memory. *Psychopharmacology (Berl)* 188 567–585. 10.1007/s00213-006-0404-5 16670842

[B14] Ge J. Wu P. Peng S. Yu H. Zhang H. Guan Y. (2015). Assessing cerebral glucose metabolism in patients with idiopathic rapid eye movement sleep behavior disorder. *J. Cereb. Blood Flow Metab.* 35:1902. 10.1038/jcbfm.2015.208 26517815 PMC4635250

[B15] Gilman S. Wenning G. K. Low P. A. Brooks D. J. Mathias C. J. Trojanowski J. Q. (2008). Second consensus statement on the diagnosis of multiple system atrophy. *Neurology* 71 670–676. 10.1212/01.wnl.0000324625.00404.15 18725592 PMC2676993

[B16] Gratwicke J. Jahanshahi M. Foltynie T. (2015). Parkinson’s disease dementia: a neural networks perspective. *Brain* 138(Pt 6) 1454–1476. 10.1093/brain/awv104 25888551 PMC4614131

[B17] Guo Q. Zhao Q. Chen M. Ding D. Hong Z. (2009). A comparison study of mild cognitive impairment with 3 memory tests among Chinese individuals. *Alzheimer Dis. Assoc. Disord.* 23 253–259. 10.1097/WAD.0b013e3181999e92 19812468

[B18] Homma T. Mochizuki Y. Komori T. Isozaki E. (2016). Frequent globular neuronal cytoplasmic inclusions in the medial temporal region as a possible characteristic feature in multiple system atrophy with dementia. *Neuropathology* 36 421–431. 10.1111/neup.12289 26970514

[B19] Jellinger K. A. (2020). Neuropathological findings in multiple system atrophy with cognitive impairment. *J. Neural Transm.* 127 1031–1039. 10.1007/s00702-020-02201-2 32367182

[B20] Kawabata K. Hara K. Watanabe H. Bagarinao E. Ogura A. Masuda M. (2019). Alterations in cognition-related cerebello-cerebral networks in multiple system atrophy. *Cerebellum* 18 770–780. 10.1007/s12311-019-01031-7 31069705

[B21] Kawai Y. Suenaga M. Takeda A. Ito M. Watanabe H. Tanaka F. (2008). Cognitive impairments in multiple system atrophy: MSA-C vs MSA-P. *Neurology* 70(16 Pt 2) 1390–1396. 10.1212/01.wnl.0000310413.04462.6a 18413566

[B22] Kim H. W. Oh M. Oh J. S. Oh S. J. Lee S. J. Chung S. J. (2017). Striatofrontal deafferentiation in MSA-P: evaluation with [18F]FDG brain PET. *PLoS One* 12:e0169928. 10.1371/journal.pone.0169928 28085923 PMC5234778

[B23] Koga S. Parks A. Uitti R. J. van Gerpen J. A. Cheshire W. P. Wszolek Z. K. (2017). Profile of cognitive impairment and underlying pathology in multiple system atrophy. *Mov. Disord.* 32 405–413. 10.1002/mds.26874 27859650 PMC5359072

[B24] Konagaya M. Sakai M. Matsuoka Y. Konagaya Y. Hashizume Y. (1999). Multiple system atrophy with remarkable frontal lobe atrophy. *Acta Neuropathol.* 97 423–428. 10.1007/s004010051008 10208284

[B25] Li N. Yang T. Ran W. Zhang X. Wang Y. Xu Z. (2021). A study on the characteristics of cognitive function in patients with multiple system atrophy in China. *Sci. Rep.* 11:4995. 10.1038/s41598-021-84393-5 33654145 PMC7925668

[B26] Lucas J. A. Ivnik R. J. Smith G. E. Ferman T. J. Willis F. B. Petersen R. C. (2005). Mayo’s older African Americans normative studies: norms for boston naming test, controlled oral word association, category fluency, animal naming, token test, WRAT-3 reading, trail making test, stroop test, and judgment of line orientation. *Clin. Neuropsychol.* 19 243–269. 10.1080/13854040590945337 16019707

[B27] Miki Y. Foti S. C. Hansen D. Strand K. M. Asi Y. T. Tsushima E. (2020). Hippocampal alpha-synuclein pathology correlates with memory impairment in multiple system atrophy. *Brain* 143 1798–1810. 10.1093/brain/awaa126 32385496

[B28] Ricci M. Pigliautile M. D’Ambrosio V. Ercolani S. Bianchini C. Ruggiero C. (2016). The clock drawing test as a screening tool in mild cognitive impairment and very mild dementia: a new brief method of scoring and normative data in the elderly. *Neurol. Sci.* 37 867–873. 10.1007/s10072-016-2480-6 26863871

[B29] Salvesen L. Winge K. Brudek T. Agander T. K. Løkkegaard A. Pakkenberg B. (2017). Neocortical neuronal loss in patients with multiple system atrophy: a stereological study. *Cereb. Cortex* 27 400–410. 10.1093/cercor/bhv228 26464477

[B30] Santangelo G. Cuoco S. Picillo M. Erro R. Squillante M. Volpe G. (2020). Evolution of neuropsychological profile in motor subtypes of multiple system atrophy. *Parkinsonism Relat. Disord.* 70 67–73. 10.1016/j.parkreldis.2019.12.010 31881520

[B31] Schmahmann J. D. Sherman J. C. (1998). The cerebellar cognitive affective syndrome. *Brain* 121(Pt 4) 561–579. 10.1093/brain/121.4.561 9577385

[B32] Shen C. Chen L. Ge J. J. Lu J. Y. Chen Q. S. He S. J. (2021). Cerebral metabolism related to cognitive impairments in multiple system atrophy. *Front. Neurol.* 12:652059. 10.3389/fneur.2021.652059 33868154 PMC8047308

[B33] Sheridan L. K. Fitzgerald H. E. Adams K. M. Nigg J. T. Martel M. M. Puttler L. I. (2006). Normative symbol digit modalities test performance in a community-based sample. *Arch. Clin. Neuropsychol.* 21 23–28. 10.1016/j.acn.2005.07.003 16139470

[B34] Siri C. Duerr S. Canesi M. Delazer M. Esselink R. Bloem B. R. (2013). A cross-sectional multicenter study of cognitive and behavioural features in multiple system atrophy patients of the parkinsonian and cerebellar type. *J. Neural Transm.* 120 613–618. 10.1007/s00702-013-0997-x 23462799

[B35] Stankovic I. Krismer F. Jesic A. Antonini A. Benke T. Brown R. G. (2014). Cognitive impairment in multiple system atrophy: a position statement by the Neuropsychology Task Force of the MDS multiple system atrophy (MODIMSA) study group. *Mov. Disord.* 29 857–867. 10.1002/mds.25880 24753321 PMC4175376

[B36] Stefanova N. Bucke P. Duerr S. Wenning G. K. (2009). Multiple system atrophy: an update. *Lancet Neurol.* 8 1172–1178. 10.1016/S1474-4422(09)70288-1 19909915

[B37] Steinberg B. A. Bieliauskas L. A. Smith G. E. Ivnik R. J. (2005). Mayo’s older americans normative studies: age- and IQ-adjusted norms for the trail-making test, the stroop test, and MAE controlled oral word association test. *Clin. Neuropsychol.* 19 329–377. 10.1080/13854040590945210 16120535

[B38] Wenning G. K. Tison F. Ben Shlomo Y. Daniel S. E. Quinn N. P. (1997). Multiple system atrophy: a review of 203 pathologically proven cases. *Mov. Disord.* 12 133–147. 10.1002/mds.870120203 9087971

[B39] Wu L. Liu F. T. Ge J. J. Zhao J. Tang Y. L. Yu W. B. (2018). Clinical characteristics of cognitive impairment in patients with Parkinson’s disease and its related pattern in (18) F-FDG PET imaging. *Hum. Brain Mapp.* 39 4652–4662. 10.1002/hbm.24311 29999569 PMC6866526

[B40] Zhao Q. Guo Q. Li F. Zhou Y. Wang B. Hong Z. (2013). The Shape Trail Test: application of a new variant of the Trail making test. *PLoS One* 8:e57333. 10.1371/journal.pone.0057333 23437370 PMC3577727

