# Supplementary material for: The Frontal and Cerebellar Metabolism Related to Cognitive Dysfunction in Multiple System Atrophy
Source: Front Aging Neurosci. 2022 Feb 10;14:788166. doi: 10.3389/fnagi.2022.788166 (PMC8871713; doi:10.3389/fnagi.2022.788166)
Supplement: Supplementary file 1 [file Table_1.DOCX]

**Supplementary Table 1**. Brain regions with significant metabolic differences between MSA-P and MSA-C patients

| Groups | Regions | | BA | MNI coordinate | | | Z max | Cluster size (mm^3^) |
| --- | --- | --- | --- | --- | --- | --- | --- | --- |
|  |  |  | | x | y | z |  |  |
| **MSA-P vs MSA-C** |  | |  |  |  |  |  |  |
| Increased metabolism | Right Nodule (Cerebellum) # | | / | 18 | -62 | -32 | 6.87 | 21952 |
|  | Right Nodule (Cerebellum) # | | / | 12 | -58 | -36 | 6.47 | 21952 |
|  | Right Inferior Semi-Lunar Lobule (Cerebellum) # | | / | 16 | -66 | -50 | 6.01 | 21952 |
|  | Left Lingual Gyrus # | | 18 | -24 | -78 | -10 | 5.46 | 4608 |
|  | Left Parahippocampal Gyrus # | | 19 | -34 | -52 | -10 | 4.70 | 4608 |
|  | Right Lingual Gyrus # | | 19 | 24 | -74 | -8 | 4.63 | 2064 |
|  | Right Declive (Cerebellum) # | | / | 32 | -60 | -12 | 4.3 | 2064 |
|  | Right Fusiform Gyrus # | | 37 | 40 | -44 | -18 | 3.24 | 2064 |
|  | Left Cuneus # | | 18 | -28 | -102 | -8 | 4.33 | 1040 |
|  | Left Cuneus # | | 18 | -16 | -106 | -2 | 4.12 | 1040 |
| Decreased metabolism | Right Lentiform Nucleus # | | Putamen | 30 | -8 | 6 | 8.08 | 36568 |
|  | Left Lentiform Nucleus # | | Putamen | -14 | 10 | 0 | 5.04 | 36568 |
|  | Left Lentiform Nucleus # | | Putamen | -24 | -6 | 8 | 5.00 | 36568 |
|  | Right Lingual Gyrus # | | 18 | 6 | -78 | -12 | 3.73 | 3256 |
|  | Right Inferior Occipital Gyrus # | | 17 | 16 | -94 | -20 | 3.25 | 3256 |

Statistical threshold: p < .001.

# indicates the cluster survived after FDR correction, p< 0.05.

Note: BA= Brodmann area; MNI = Montreal Neurological Institute; MSA-P = Multiple system atrophy with predominant parkinsonism; MSA-C = MSA with predominant cerebellar ataxia; FDR = False Discovery Rate.

**Supplementary Table 2**. Brain regions in separate MSA-P and MSA-C patients exhibiting positive correlations between the Z-score of cognitive domains and regional brain metabolism adjusting for education and UPDRS score

| MSA subtypes | Domains | Regions | BA | MNI coordinate | | | Z max | Cluster size (mm^3^) |
| --- | --- | --- | --- | --- | --- | --- | --- | --- |
|  |  |  |  | x | y | z |  |  |
| **MSA-P** | Attention | Right Tuber (Cerebellum) | / | 32 | -60 | -40 | 4.62 | 8408 |
|  |  | Right Declive (Cerebellum) | / | 4 | -64 | -28 | 3.95 | 8408 |
|  |  | Right Uvula (Cerebellum) | / | 10 | -62 | -40 | 3.90 | 8408 |
|  | Language | Right Cingulate Gyrus | 24 | 6 | -8 | 34 | 4.77 | 1728 |
| **MSA-C** | Attention | Left Inferior Frontal Gyrus # | 45 | -56 | 18 | 4 | 6.24 | 7696 |
|  |  | Left Inferior Frontal Gyrus # | 9 | -48 | 8 | 26 | 3.93 | 7696 |
|  |  | Left Inferior Frontal Gyrus # | 45 | -50 | 18 | 22 | 3.84 | 7696 |
|  |  | Left Thalamus # | / | -8 | -16 | 8 | 5.18 | 2488 |
|  |  | Left Medial Frontal Gyrus # | 9 | -4 | 44 | 38 | 4.60 | 6032 |
|  |  | Left Anterior Cingulate # | 32 | -4 | 38 | 18 | 4.52 | 6032 |
|  |  | Left Anterior Cingulate # | 24 | -4 | 28 | 24 | 4.36 | 6032 |
|  |  | Left Superior Frontal Gyrus # | 8 | -16 | 26 | 52 | 4.59 | 6800 |
|  |  | Left Superior Frontal Gyrus | 10 | -18 | 66 | -8 | 4.40 | 1000 |
|  |  | Left Middle Frontal Gyrus # | 8 | -26 | 22 | 48 | 4.44 | 6800 |
|  |  | Left Middle Frontal Gyrus # | 6 | -46 | 2 | 50 | 4.35 | 6800 |
|  |  | Right Inferior Frontal Gyrus # | 44 | 66 | 12 | 14 | 4.25 | 2880 |
|  |  | Right Inferior Frontal Gyrus # | 47 | 52 | 18 | -4 | 4.15 | 2880 |
|  |  | Right Tuber (Cerebellum) # | / | 36 | -74 | -38 | 4.20 | 1064 |
|  | Executive function | Right Tuber (Cerebellum) | / | 34 | -70 | -38 | 3.97 | 1400 |
|  |  | Right Tuber (Cerebellum) | / | 38 | -62 | -38 | 3.80 | 1400 |
|  |  | Right Pyramis (Cerebellum) | / | 22 | -74 | -38 | 3.79 | 1400 |
|  |  | Left Medial Frontal Gyrus | 6 | -22 | 2 | 54 | 3.93 | 1328 |
|  |  | Left Superior Frontal Gyrus | 6 | -12 | 10 | 62 | 3.86 | 1328 |
|  |  | Left Claustrum | / | -36 | 4 | -8 | 3.83 | 1024 |
|  | Language | Left Middle Frontal Gyrus | 8 | -26 | 20 | 40 | 4.21 | 1600 |
|  |  | Right Tuber (Cerebellum) | / | 36 | -72 | -38 | 3.86 | 1136 |
|  |  | Right Tuber (Cerebellum) | / | 38 | -60 | -38 | 3.73 | 1136 |
|  | Memory | Left Middle Frontal Gyrus | 8 | -28 | 20 | 46 | 4.19 | 1152 |

Statistical threshold: p < .001.

# indicates the cluster survived after FDR correction, p< 0.05. Note: BA= Brodmann area; MNI = Montreal Neurological Institute; MSA-P = Multiple system atrophy with predominant parkinsonism; MSA-C = MSA with predominant cerebellar ataxia; FDR = False Discovery Rate.

**Supplementary Table 3**. Brain regions in separate MSA-P and MSA-C patients exhibiting negative correlations between the Z-score of cognitive domains and regional brain metabolism adjusting for education and UPDRS score

| MSA subtypes | Domains | Regions | BA | MNI coordinate | | | Z max | Cluster size (mm^3^) |
| --- | --- | --- | --- | --- | --- | --- | --- | --- |
|  |  |  |  | x | y | z |  |  |
| **MSA-P** | Attention | Left Superior Temporal Gyrus | 38 | -34 | 10 | -30 | 4.42 | 1024 |
|  | Executive function | Left Superior Temporal Gyrus | 38 | -32 | 12 | -36 | 5.94 | 3224 |
|  |  | Left Parahippocampal Gyrus | / | -34 | -6 | -30 | 3.95 | 3224 |
|  |  | Right Uncus | 20 | 34 | 2 | -38 | 4.25 | 1096 |
|  |  | Right Superior Temporal Gyrus | 38 | 40 | 10 | -34 | 3.76 | 1096 |
| **MSA-C** | Attention | Right Insula | 13 | 50 | 26 | -26 | 4.22 | 4048 |
|  |  | Right Postcentral Gyrus | 2 | 40 | -28 | 28 | 4.12 | 4048 |
|  |  | Right Insula | 13 | 42 | -12 | 26 | 4.09 | 4048 |
|  |  | Right Insula | 19 | 50 | -74 | -22 | 4.19 | 1240 |
|  | Memory | Right Fusiform Gyrus | 19 | 46 | -80 | -22 | 4.31 | 1048 |

Statistical threshold: p < .001.

# indicates the cluster survived after FDR correction, p< 0.05. Note: BA= Brodmann area; MNI = Montreal Neurological Institute; MSA-P = Multiple system atrophy with predominant parkinsonism; MSA-C = MSA with predominant cerebellar ataxia; FDR = False Discovery Rate.

.
